# A randomized controlled trial comparing in-person and wiki-inspired nominal group techniques for engaging stakeholders in chronic kidney disease research prioritization

**DOI:** 10.1186/s12911-016-0351-y

**Published:** 2016-08-24

**Authors:** Meghan J. Elliott, Sharon E. Straus, Neesh Pannu, Sofia B. Ahmed, Andreas Laupacis, George C. Chong, David R. Hillier, Kate T. Huffman, Andrew C. Lei, Berlene V. Villanueva, Donna M. Young, Helen Tam-Tham, Maoliosa Donald, Erin Lillie, Braden J. Manns, Brenda R. Hemmelgarn

**Affiliations:** 1 Li Ka Shing Knowledge Institute, St. Michael’s Hospital, 209 Victoria Street, East Building, Toronto, Ontario M5B 1T8 Canada; 2 Institute of Health Policy, Management and Evaluation, University of Toronto, 155 College Street, Toronto, Ontario M5T 3M6 Canada; 3 Department of Medicine, University of Toronto, Suite RFE 3-805, 200 Elizabeth Street, Toronto, Ontario M5G 2C4 Canada; 4 Department of Medicine, University of Alberta, 8440 112 Street, Edmonton, Alberta T6G 2G3 Canada; 5 Department of Medicine, University of Calgary, 1403 29 Street NW, Calgary, Alberta T2N 2T9 Canada; 6 Suite 200, 5970 Centre Street SE, Calgary, Alberta T2H 0C1 Canada; 7 343 Webb Court, Newmarket, Ontario L3Y 5E8 Canada; 8 23-1140 Falcon Drive, Coquitlam, British Columbia V3E 2J6 Canada; 9 4K Spadina Avenue, Suite 1820, Toronto, Ontario M5V 3Y9 Canada; 10 A4123-409 Tache Avenue, Winnipeg, Manitoba R2H 2A6 Canada; 11 31 Seligs Road, Prospect Village, Nova Scotia B3T 2A6 Canada; 12 Department of Community Health Sciences, University of Calgary, 3280 Hospital Drive NW, Calgary, Alberta T2N 4Z6 Canada

**Keywords:** Chronic kidney disease, Research priorities, Patient preferences, Wiki

## Abstract

**Background:**

Few studies have evaluated stakeholder engagement in chronic kidney disease (CKD) research prioritization. In this two-arm, parallel group randomized controlled trial, we sought to compare an in-person nominal group technique (NGT) approach with an online wiki-inspired alternative to determining the top 10 CKD research priorities, and to evaluate stakeholder engagement and satisfaction with each process.

**Methods:**

Eligible participants included adults ≥18 years with access to a computer and Internet, high health literacy, and from one of the following stakeholder groups: patients with CKD not on dialysis, their caregivers, health care providers who care for patients with CKD, or CKD-related health policymakers. Fifty-six participants were randomized to a wiki-inspired modified NGT that occurred over 3 weeks vs. a 1-day in-person NGT workshop, informed by James Lind Alliance methodology, to determine the top 10 CKD-related research priorities. The primary outcome was the pairwise agreement between the two groups’ final top 10 ranked priorities, evaluated using Spearman’s correlation coefficient. Secondary outcomes included participant engagement and satisfaction and wiki tool usability.

**Results:**

Spearman’s rho for correlation between the two lists was 0.139 (95 % confidence interval −0.543 to 0.703, *p* = 0.71), suggesting low correlation between the top 10 lists across the two groups. Both groups ranked the same item as the top research priority, with 5 of the top 10 priorities ranked by the wiki group within the top 10 for the in-person group. In comparison to the in-person group, participants from the wiki group were less likely to report: satisfaction with the format (73.7 vs.100 %, *p* = 0.011); ability to express their views (57.9 vs 96.0 %, *p* = 0.0003); and perception that they contributed meaningfully to the process (68.4 vs 84.0 %, *p* = 0.004).

**Conclusions:**

A CKD research prioritization approach using an online wiki-like tool identified low correlation in rankings compared with an in-person approach, with less satisfaction and perceptions of active engagement. Modifications to the wiki-inspired tool are required before it can be considered a potential alternative to an in-person workshop for engaging patients in determining research priorities.

**Trial registration:**

(ISRCTN18248625)

**Electronic supplementary material:**

The online version of this article (doi:10.1186/s12911-016-0351-y) contains supplementary material, which is available to authorized users.

## Background

Chronic kidney disease (CKD), defined as estimated glomerular filtration rate (eGFR) <60 mL/min/1.73 m^2^, affects approximately 5–7 % of the adult population [1–3] and contributes to excess morbidity, mortality and health care costs [4, 5]. Despite the tremendous burden CKD places on health care delivery, care for persons affected by CKD remains suboptimal [6]. These care gaps may result from the lack of research that is relevant to end-users, including patients with CKD, their caregivers, and the clinicians involved in their care [7, 8]. To address these gaps, there is increasing emphasis on the importance of engaging key stakeholders in determining research priorities and establishing user-relevant research objectives [9, 10]. The Strategy for Patient Oriented Research (SPOR) [11] in Canada and the Patient-Centered Outcomes Research Institute (PCORI) in the United States [12] are two initiatives that aim to engage patients in research and knowledge translation and to focus on interventions and outcomes that are important to research end users.

Although few established methods for involving key stakeholders in research prioritization exist, the James Lind Alliance (JLA) priority setting partnership model is widely considered an inclusive and transparent process and has been applied to a number of disease conditions [13]. The final step of the four-step JLA partnership involves an in-person workshop with a nominal group technique (NGT) to use a list of the top 30 research priorities to develop a ranked list of the top 10 research priorities from key stakeholders through small and large group activities [14]. While considered the reference standard, this approach has some clear limitations for participants and coordinators, including time and resource requirements, travel, and logistical consideration [15]. These challenges may be particularly problematic for patients with chronic illness who have difficulty with or are unable to travel, which could limit participant involvement and generalizability of findings. Alternative Internet-based approaches to research priority setting, such as an interactive wiki-like platform, could obviate some of these limitations. A ‘wiki’ is a collaborative web-based platform that allows users to add and edit content online in real time, and in the context of research prioritization can be used to summarize the preferences of multiple stakeholders [16]. Wiki-inspired tools have been successfully applied in similar, multi-stakeholder consensus approaches, including the creation of an asthma action plan [16], but have not been studied in research prioritization exercises that require significant patient involvement.

In this randomized controlled trial we sought to compare the in-person workshop and NGT approach with an online wiki-inspired alternative as the final step in the JLA research prioritization process for key CKD stakeholders, including patients with CKD not on dialysis, their caregivers, and the health care professionals and policymakers involved in their care. We aimed to compare the top 10 research priorities resulting from each process and to evaluate the engagement and satisfaction of participating stakeholders with their assigned intervention.

## Methods

This trial is reported in accordance with the Consolidated Standards of Reporting Trials (CONSORT) 2010 statement [17] (trial registration: www.isrctn.org, ISRCTN18248625).

### Overview of the JLA CKD priority setting partnership

The JLA priority setting partnership uses established methods to identify and prioritize unanswered disease- or treatment-related questions [14]. Our CKD priority setting process involved: 1) creation of a 12-person steering group of patients with non-dialysis CKD, caregivers, clinicians and researchers; 2) identification of research uncertainties through a national survey of representative CKD stakeholders; 3) refinement and prioritization of the research uncertainties identified in the survey to determine a shortlist of the top 30 research priorities by the steering group; and, 4) participation by stakeholders from across Canada in a 1-day priority setting workshop to determine the top 10 CKD research priorities from a shortlist of 30. Steps 1–3 took place from July 2014 to June 2015; this trial involved step 4 only.

### Trial participants

The steering group enlisted the support of nephrology networks, partner organizations, and nephrologists from across Canada to identify potential participants. Eligible participants were ≥18 years of age, English speaking, had access to and were comfortable using high-speed Internet, and had high health literacy (determined by asking the following question: “How confident are you in filling out medical forms by yourself?” [18]). Participants were also required to be a member of a stakeholder group: patients with CKD not on dialysis or with a prior transplant; informal caregivers of persons with CKD (relatives, family members, or friends who help patients manage their illness); health care professionals (primary care physicians, nephrologists, nurses, pharmacists, social workers, or dietitians) who care for patients with CKD; or health policymakers (non-clinicians with the ability to influence or determine policies and practices related to health care delivery for CKD at an international, national, regional, or local level). The following individuals were not eligible to participate: patients receiving dialysis and/or who had received a kidney transplant; persons with an underlying diagnosis of dementia or cognitive impairment; and patients deemed unfit to travel. A research assistant confirmed the eligibility of potential participants, described the trial to them in detail, and obtained their written informed consent.

### Interventions

Participants were randomized 1:1 to an in-person NGT-based workshop or an online wiki-inspired platform. Randomization occurred by random number sequence generation using a central electronic system to ensure allocation concealment. Patients and their caregivers were randomized as a dyad to avoid contamination. Additionally, the 6 patient and caregiver steering group members were allocated to the in-person workshop group only, and not randomized, as they had originally committed to participate in the NGT process of the study and it was therefore deemed important to include them in the NGT arm. Participants in both groups were provided with the top 30 uncertainties shortlist (derived from a national survey and prioritized by the steering group) to review and consider prior to the intervention; participants in the in-person workshop group ranked the 30 uncertainties prior to the workshop, while participants in the wiki-inspired group ranked the top 10 (due to logistical issues of ranking 30 online).

Individuals in the 1-day in-person workshop group participated in a NGT approach [14, 19] in Toronto, Canada, which included a combination of small and large group exercises facilitated by four individuals with experience in the JLA process. The final ranking of the top 10 research priorities was determined by large group consensus, with voting when needed.

Wiki group participants were provided with a 1-h training webinar on use of the wiki and were given unique usernames and passwords to access the secure website before they began the following tasks to determine the top 10 research priorities. The wiki-based intervention took place online over 3 phases: 1) an individual ranking phase (7 days), where participants submitted their preferences for the top 10 research priorities individually, 2) a group ranking phase (12 days), where participants determined the final top 10 research priorities collaboratively, and 3) a sign-off phase (2 days), where participants could review to confirm their agreement, but not re-order the final list. Following the individual phase, individual rankings were aggregated across participants using reverse scoring, such that items ranked first received a score of 10, and those ranked last received a score of 1. These scores were aggregated and presented as a summary rank list to which participants could refer throughout the collaborative ranking phase. During the collaborative ranking phase, participants could re-order the top 10 priorities in real time and discuss their selections using a chat feature. The chat feature consisted of a text box and display of all participant comments in chronological order, organized by unique user identifier (ie identifying information and stakeholder role were not apparent during chat to avoid differential perception of input by provider role). A facilitator monitored the site daily and stimulated discussion through comments using the chat feature. Technical support was available throughout the process. Due to the nature of the interventions, study participants and intervention administrators were not blinded to group assignment; however, statistical analysis was blinded. The in-person workshop took place during the wiki process; participants in both groups were blinded to the ranking results of the other group.

### Outcomes

The primary outcome was the pairwise agreement between the two groups’ final top 10 ranked priorities recorded upon conclusion of the workshop and group ranking phase for the in-person and wiki groups, respectively. Secondary outcomes included perceived engagement of participants in their assigned prioritization process and participant satisfaction. We also evaluated usability aspects of the online wiki tool including content, features, and accessibility (see Additional file 1 for details on outcomes and methods of measurement). The electronic questionnaire assessing participant engagement and satisfaction and wiki usability was administered using the FluidSurveys platform (FluidSurveys platform, Fluidware Inc., Ottawa, Ontario)., Key elements of stakeholder engagement were evaluated in the participant engagement section of the questionnaire, including trust, respect, accountability, legitimacy, fairness, and competence [20, 21].

### Sample size

The primary outcome was the pair wise agreement between the two groups’ top 10 priorities, therefore the correlation analysis was driven by the number of research priorities considered, which was fixed at 10 for the purposes of this study. As the JLA recommends 20 to 30 participants in the in-person priority setting exercise, with approximately equal representation of patients, clinicians, and caregivers, we estimated that a total of 40–60 participants would be required (we aimed for 50). Inflating this by 10 % to account for potential dropouts, an overall; sample size of 56 was felt to be a conservative estimate for the primary outcome.

### Statistical analyses

Baseline demographic information was compared between the in-person workshop and wiki groups using univariate analyses. The unit of analysis for the primary outcome was at the group level, and the pairwise agreement between the two groups’ priorities (provided as ranks) was examined using Spearman’s correlation coefficient and its associated 95 % confidence interval (CI). We also compared the rank order of the top 10 research priorities from each group descriptively, noting the proportion that were within the top 10 across both groups. Our secondary outcomes were obtained by survey for both the in-person and wiki groups, with responses measured using a 5-point Likert scale (where 1 = strongly disagree, 5 = strongly agree), and the proportions of participants who agreed with each statement (4 or 5 on the scale) were compared between groups using chi-square tests. Written comments also obtained from the survey by both groups were examined qualitatively. Two researchers (MJE and BRH) reviewed all written comments and independently coded them for emerging categories and themes. Resulting themes and discrepancies were discussed, and a final coding scheme describing participants’ experiences with each process was established. Participant observation data collected during the in-person workshop was summarized quantitatively (ie frequencies) and qualitatively (ie brief researcher notes). The time requirements to undertake each process were compared descriptively.

## Results

### Participants

**Fig. 1:**
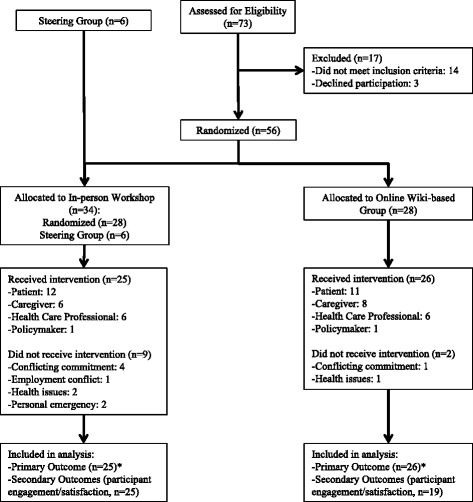
Flow diagram outlining intervention group allocation and withdrawals. *Primary outcome measured at the group level

Fifty-six individuals were randomized to either the in-person workshop (*n* = 28) or online wiki-inspired group (*n* = 28). In addition the 6 patient and caregiver steering group members were allocated to the in-person workshop group only, as established a priori, for a total of 34 participants for the in-person workshop. Overall 25 individuals took part in the 1-day workshop (12 patients, 6 caregivers, 6 health care professionals, and 1 policymaker), and 26 individuals participated in the 3-week online wiki-like process (12 patients, 8 caregivers, 5 health care professionals, and 1 policymaker). Participant enrolment and reasons for withdrawal are outlined in Fig. 1. During the 3-week period of wiki activity, 21 of 26 participants (80.8 %) logged onto the ranking page at least once during either the individual or group phase (3 patients and 2 caregivers did not access the site during the intervention). All participants contributed to the primary analysis of agreement between the two top 10 research priority lists, as this was assessed at the group level. Only participants who completed the post-intervention survey were included in the analysis of participant engagement and satisfaction.

The majority of study participants were 40–64 years of age, and 47 % were male (Table 1). 12 patients and 6 caregivers participated in the in-person workshop, and 11 patients and 8 caregivers participated in the online wiki-inspired process. Compared to the in-person group, a smaller proportion of patients and caregivers in the wiki-inspired group were < 40 years of age and female (5.6 vs. 21.1 % and 38.9 vs 52.6 % respectively), and they were less likely to work full time (31.6 vs. 44.4 %) or hold a university degree (26.3 vs. 61.1 %).

**Table 1:** Baseline demographics of participants in the in-person workshop and online wiki groups, n (%)

	In-person workshop group (*n* = 25)	Patients and caregivers in in-person workshop (*n* = 18)	Online wiki-inspired group (*n* = 26)	Patients and caregivers in online wiki-inspired group (*n* = 19)
Age				
18–39 years	3 (12)	1 (5.6)	4 (15.4)	4 (21.1)
40–64 years	14 (56)	9 (50)	16 (61.5)	9 (47.4)
≥ 65 years	8 (32)	8 (44.4)	6 (23.1)	6 (31.6)
Sex				
Male	13 (52)	11 (61.1)	11 (42.3)	9 (47.4)
Female	12 (48)	7 (38.9)	15 (57.7)	10 (52.6)
Role				
Patient	12 (48)	12 (66.7)	11 (42.3)	11 (57.9)
Caregiver	6 (24)	6 (33.3)	8 (30.8)	8 (42.1)
Health Care Provider	6 (24)	-	6 (23.1)	-
Policymaker	1 (4)	-	1 (3.8)	-
Marital Status				
Married	22 (88)	16 (88.9)	19 (73.1)	13 (68.4)
Common Law	1 (4)	0 (0)	2 (7.7)	1 (5.3)
Divorced/Separated	2 (8)	2 (11.1)	0 (0)	0 (0)
Single	0 (0)	0 (0)	3 (11.5)	3 (15.8)
Not Indicated	0 (0)	0 (0)	2 (7.7)	2 (10.5)
Employment Status				
Full-time	14 (56)	8 (44.4)	12 (46.2)	6 (31.6)
Part-time or casual	2 (8)	1 (5.6)	2 (7.7)	1 (5.3)
Retired	9 (36)	9 (50)	10 (38.5)	10 (52.6)
Unemployed	0 (0)	0 (0)	1 (3.8)	1 (5.3)
Not Indicated	0 (0)	0 (0)	1 (3.8)	1 (5.3)
Highest Level of Education				
Some High School	0 (0)	0 (0)	1 (3.8)	1 (5.3)
High School Graduate	4 (16)	4 (22.2)	3 (11.5)	3 (15.8)
College Diploma	4 (16)	3 (16.7)	8 (30.8)	8 (42.1)
University Degree	17 (68)	11 (61.1)	12 (46.2)	5 (26.3)
Not Indicated	0 (0)	0 (0)	2 (7.7)	2 (10.5)

### Comparison of the top 10 CKD research priorities

At the conclusion of the in-person workshop and wiki-inpsired interventions, the top 10 research priorities were determined for each group (Table 2). Overall, Spearman’s rho for correlation between the two groups was 0.139 (95 % CI −0.543 to 0.703, *p* = 0.71), suggesting a non-significant low level of correlation between the two groups. Both groups independently ranked the same item as the top research priority (*What are the most effective new interventions and treatments to prevent the development and progression of kidney disease?*). Of the top 10 research priorities ranked by the wiki-inspired group, 5 were ranked within the top 10 by the workshop group, and a further 4 items were ranked within the top 15 by the workshop group. Of note, 5 of the top 10 research priorities in the NGT group were not in the top 10 of the wiki-inspired group, with four of these being health services research questions. The #10 priority in the NGT workshop group was extensively discussed in both small and large group settings, suggesting that greater interaction in the NGT workshop setting may have influenced the final rankings. The research priorities that were common to the top 10 lists of both groups included dietary measures to slow CKD progression, CKD symptoms, and the impact of lifestyle factors and medications on kidney disease. Two wiki group members (both clinicians) had participated previously in an in-person priority setting workshop for patients on or nearing dialysis [22], which may have influenced their perceptions and experiences of the intervention.

**Table 2:** Top 10 CKD-related research priorities resulting from the in-person workshop vs. online wiki-based groups

In-person workshop group	Wiki rank	Online wiki-based group	Workshop rank
1. What are the most effective new interventions and treatments to prevent the development and progression of kidney disease?	1	1. What are the most effective new interventions and treatments to prevent the development and progression of kidney disease?	1
2. What is the best diet to slow progression of kidney disease and what are the benefits and risks of specific diets (ie phosphate restriction, protein restriction, low salt etc.) in terms of kidney disease progression and quality of life?	6	2. What are the harmful effects of medications used in patients with CKD, and in particular, the combinations of medications used to treat other diseases (such as diabetes and high blood pressure)?	7
3. What are the causes of symptoms in patients with chronic kidney disease, including fatigue, low energy, sleeping problems, depression, anxiety and sexual dysfunction, and how can these best be treated to improve quality of life?^a^	8	3. What are the best signs or markers (ie blood tests, urine tests or other tests) to identify and diagnose kidney disease early?	14
4. What are the optimal strategies, such as having access to health information (eg lab test results), sharing of information, and/or improving communication, to help patients manage their health condition(s) themselves and to improve patient experience and outcomes?	Not ranked in the top 10	4. What are the benefits and risks associated with use of vitamins, supplements and alternative/complementary therapies (ie herbal, naturopathic, marijuana etc.) in terms of kidney disease progression and quality of life?	11
5. What is the impact of lifestyle factors (ie exercise, stress) on risk of developing kidney disease, kidney disease progression, and quality of life?	9	5. How can we predict how fast kidney function will get worse, and when kidneys will fail?	13
6. What are the optimal strategies for the management of CKD (ie those undertaken by the primary care physician, nephrologist, other health care professionals) to delay progression and improve outcomes?	Not ranked in the top 10	6. What is the best diet to slow progression of kidney disease and what are the benefits and risks of specific diets (ie phosphate restriction, protein restriction, low salt etc.) in terms of kidney disease progression and quality of life?	2
7. What are the harmful effects of medications used in patients with CKD, and in particular the combinations of medications used to treat other diseases (such as diabetes and high blood pressure)?	2	7. What are the optimal medications (eg ACE inhibitors, ARBs, phosphate binders, sodium bicarbonate, etc.) to slow progression of kidney disease?	19
8. What are the optimal approaches for the prevention and treatment of cardiovascular disease in patients with CKD?	Not ranked in the top 10	8. What are the causes of symptoms in patients with chronic kidney disease, including fatigue, low energy, sleeping problems, depression, anxiety and sexual dysfunction, and how can these best be treated to improve quality of life?^a^	3
9. What is the best strategy (eg screening, programs targeting high risk groups, programs to increase public awareness) to identify kidney disease early?	Not ranked in the top 10	9. What is the impact of lifestyle factors (ie exercise, stress) on risk of developing kidney disease, kidney disease progression, and quality of life?	5
10. How do we ensure that patients with CKD have equitable access to care (eg nephrologists, allied health clinics) irrespective of location of residence or socio-economic status?	Not ranked in the top 10	10. How can communication regarding patient care be improved and/or streamlined across all disciplines (primary care, nephrology, allied health) to improve outcomes and the patient experience?	12

*CKD* chronic kidney disease, *ACE* angiotensin-converting enzyme, *ARB* angiotensin II receptor blockers

^a^Item subsumed a second uncertainty noted within the top 30 uncertainties in both the in-person and wiki groups

### Participant satisfaction and engagement

**Fig. 2:**
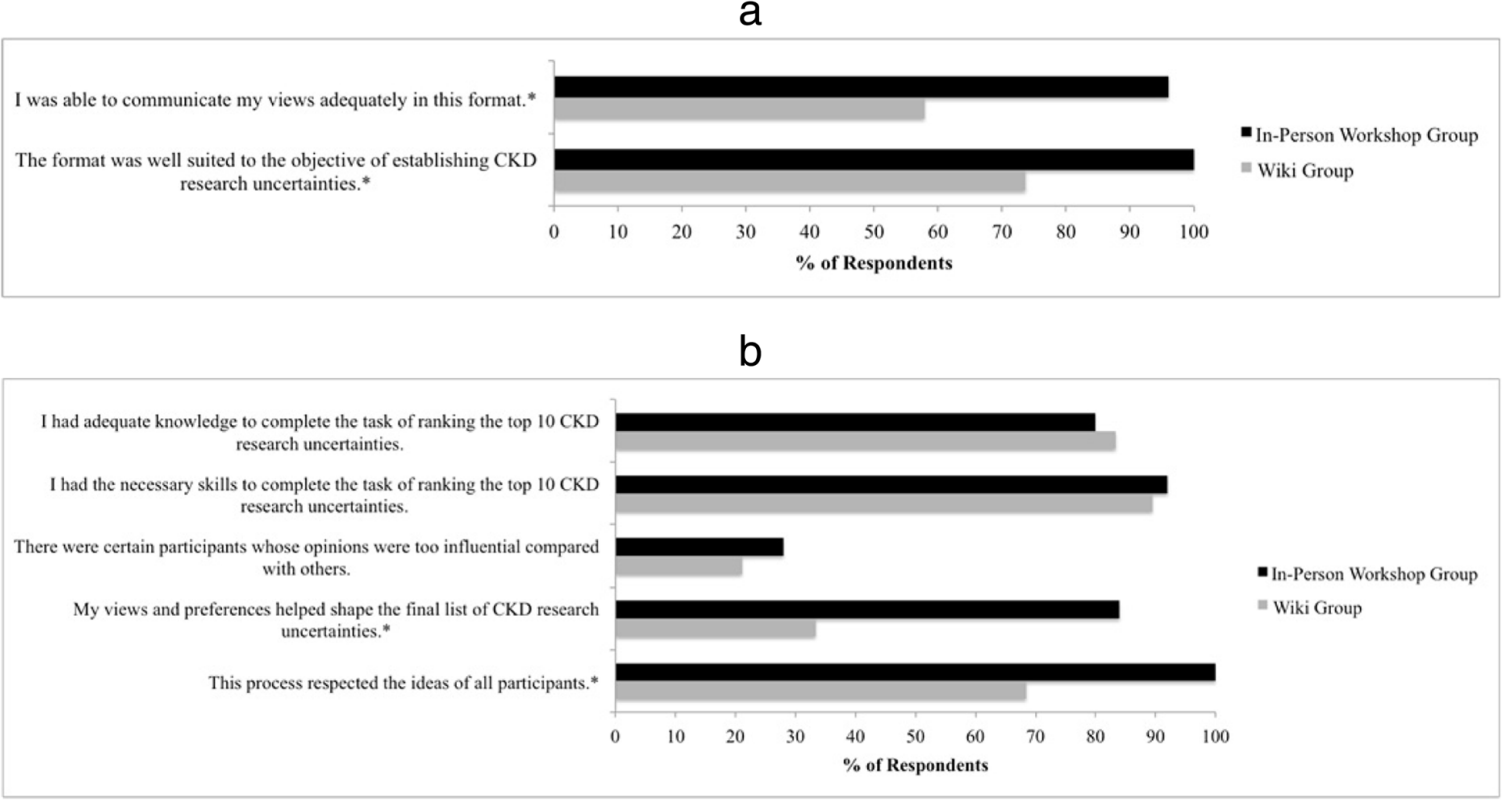
**a** Satisfaction of workshop and wiki participants on post-intervention questionnaire. *p < 0.05 for between-group difference. **b** Engagement of workshop and wiki participants on post-intervention questionnaire. *p < 0.05 for between-group difference

All 25 participants in the NGT-based workshop group and 19 of 26 participants from the online wiki-inspired group completed the post-intervention questionnaire. The non-responders from the wiki group included 4 patients, 2 caregivers and 1 health care professional. All workshop participants felt the in-person NGT format was well-suited (ie noted agreement or strong agreement) to determine CKD research uncertainties, whereas 73.7 % of respondents in the wiki group were satisfied (agree/strongly agree) with the format (*p* = 0.011) (Fig. 2a). Ninty-six percent of participants in the workshop group felt they could adequately express their views in this format, as compared to 57.9 % in the wiki group (*p* = 0.003). With respect to participant engagement, workshop participants were significantly more likely than wiki participants to report that the format used by the group respected the ideas of all participants (100 vs. 68.4 %, *p* = 0.004) and perceived that their views contributed to the final list of research priorities (84.0 vs. 33.3 %, *p* < 0.001) (Fig. 2b). Respondents from both groups felt they had adequate knowledge (80.0 % in workshop group vs. 83.3 % in wiki group, *p* = 1.0) and skills (92.0 % in workshop group vs. 89.5 % in wiki group, *p* = 1.0) to rank the top research priorities, and around one quarter of participants felt that influential participants impacted the group dynamics (28.0 % in workshop vs. 21.1 % in wiki group *p* = 0.73).

Written comments were provided in the survey by 11 participants in the in-person NGT group and 10 participants in the wiki-inspired group. The main themes that emerged were related to opportunities for personal interactions and meaningful contributions, participation rates, format effectiveness and the importance of communication to justify individual rankings (Table 3), with the workshop group generally providing more positive feedback of their experience across these three themes. Written comments from wiki participants highlighted their perceptions of a low level of active participation and inadequacy of the chat feature (which aimed to allow comprehensive discussion of participants’ views, preferences and justification of changes to the their rankings). Wiki participants felt that the top ranked uncertainties were ordered and re-ordered without a clear understanding of the reasons for the changes and with limited group discussion. In contrast, in-person workshop participants felt they were able to contribute equally and effectively among all stakeholders in this format.

**Table 3:** Main themes identified from written feedback on post-intervention questionnaire

Group	Role	Comment
Limited Personal Interactions		
Wiki	Caregiver	“It was easier not to put your whole heart into this as it would have been if it were done face to face with the other participants… I felt something was missing, some sort of connection with others facing the same difficulties.”
Wiki	Health Care Professional	“The wiki seemed to reward the last person to change the rankings. In person, a doc such as me could have had a more subtle way to influence the process without being aggressive or trying to take it over.”
Importance of Communication to Justify Selections		
Wiki	Patient	“When people explained their rationale for their preferences, I understood. When the ranking was changed without using the chat feature to augment our understanding, I could not discern the rationale for the preferences.”
Wiki	Patient	“Although I enjoyed the discussions that did occur and the mix of group members (caregiver, patient, clinician), I'm not sure, as a whole, we really made the most of the chat feature.”
Wiki	Health Care Professional	“I think any changes in the top 10 should have had comments to support these changes.”
Wiki	Health Care Professional	“In a discussion forum online, I also didn't feel comfortable asking specific people to clarify or explain their choices.”
Format Effectiveness		
Workshop	Caregiver	“The format enabled patients and caregivers to engage with a wide group of professionals, other than the traditional doctor-patient relationship.”
Wiki	Patient	“Basically, I felt this process was less effective and that we didn't actually reach a group consensus with our outcomes.”
Workshop	Patient	“The only part that was not entirely satisfactory was the final phase in which the entire group worked to re-order priorities. I felt that it lacked the fluidity and finesse to allow for minor changes.”
Perspectives Dependent on Participation		
Wiki	Patient	“I was disappointed by the participation rate (those who did not participate). I think this affected the quality of the discussions and possibly weakened the final outcome.”
Workshop	Health Care Professional	“Some opinions/voices might not be represented depending on who attends (is able to attend) and who is approached to attend the workshop.”
Ability to Contribute Meaningfully		
Workshop	Caregiver	“As a layperson I felt that even though I did not have the hard earned scientific expertise of the medical community, all our experiences were considered on their merits.”
Workshop	Patient	“The workshop was an amazing experience where a collaborative effort of all affected people by CKD joined together to come up with the top 10 important and highly timely research uncertainties that will give better life with the patients.”
Workshop	Patient	“I left feeling that in some small way I had contributed to research possibilities for my disease which currently has no cure.”

Observation of participants during the in-person workshop revealed a moderate to high degree of verbal and physical engagement for the majority of group members. Three participants (2 patients and 1 caregiver) demonstrated limited verbal and physical interaction with others. Most individuals appeared to readily voice their views, questions and comments spontaneously or with prompting by the facilitator. While the initial small group discussions were reserved and initiated mainly by the facilitator, more spontaneous and animated discussions among the participants arose throughout the day. Some participants contributed more confidently and persuasively than others, but these individuals were not limited to one stakeholder group and did not appear to negatively dominate any of the group discussions. In the wiki-inspired group, chat discussions were similarly reserved and required facilitator prompting initially. However, throughout the process, participants provided spontaneous input and sought each other’s views in a respectful manner.

### Wiki usability

During the 3-week period of wiki activity, 21 of 26 participants (80.8 %) logged onto the ranking page at least once during either the individual or group phase. Those who did not log in during the intervention included 3 patients and 2 caregivers. During the group ranking phase, 13 users (50.0 %) were considered active participants (ie they made direct changes to the top 10 uncertainties list [*n* = 11, 42.3 %] and/or participated in the chat feature [*n* = 13, 50.0 %]). These active participants included 6 patients, 3 caregivers, and 4 health professionals. One patient who was admitted to hospital during the individual ranking phase accessed the ranking tool, submitted her individual ranking, and participated in the group ranking and discussion from a laptop computer while in hospital.

Over the 12-day group ranking phase, a total of 192 changes were made to the rankings (mean 16 changes/day, range 1–37) and 104 comments were provided in the group chat (mean 8.7 comments/day, range 2–21). The most changes were made on the first day of the group round (37 changes). Twenty-six changes were made to the list on the final day, and the most group discussion (21 comments) occurred on the final day of the exercise. The most active participants included 2 patients and 1 health care provider, each of whom provided between 46 and 58 contributions to the rankings and/or group chat. Half of the wiki-inspired group participants viewed and confirmed they had reviewed the final top 10 list following conclusion of the group ranking phase.

Results from the wiki usability portion of the post-intervention questionnaire are outlined in Additional file 2. Of the 18 respondents, all felt that 12 days were sufficient to undertake the group ranking process, and 61 % used the tool as often as they would have liked. Eighty-three percent of participants experienced no difficulties in changing the rankings, and 61 % found the chat feature useful for discussing the uncertainties as a group. Six participants contacted study coordinators regarding technical issues, all of which related to difficulty logging onto the site. All issues but one were resolved, with this user being able to login after repeated attempts but having ongoing difficulties participating in the ranking process.

### Time requirements

The in-person workshop intervention took place over 1 weekday and required travel time for participants from across Canada to attend. The online wiki-based group could access the ranking site at any time of day, from any location, and as often as participants preferred during a 3-week period.

## Discussion

In this randomized trial evaluating two methods of stakeholder engagement to determine CKD research priorities, we found that an online wiki-inspired approach had a low level of correlation compared to the research priorities determined from an in-person NGT approach informed by JLA methodology, with only 5 of the top 10 priorities identified by the wiki-inspired group within the top 10 priorities of the in-person group. There were also important differences between the groups, with fewer health-services research questions ranked by the wiki-inspired group, and differences in perceptions of engagement. In comparison to participants in the in-person workshop, those in the wiki-inspired group were less satisfied with the format of the exercise and felt they were less likely to contribute meaningfully to the process. Whereas all individuals who attended the in-person workshop contributed to the group discussions (either spontaneously or with prompting), 80 % of individuals in the wiki group accessed the tool at least once, and only half were considered ‘active’ participants. We are unable to determine if the differences in research priorities identified were due to the different processes, or differences in the groups themselves (including the patient and caregiver steering group members participating in the in-person workshop), as priorities may vary by group irrespective of the process used to establish them. This will be important to address in future work.

Our study is one of the first to evaluate an online wiki-based tool to engage patients and other stakeholders in health care research prioritization and to undertake a randomized comparison of two distinct research prioritization methods. Although both techniques are based on a NGT approach, the online wiki-inspired option had attractive features including flexibility to contribute at a convenient time during a process that occurred over a longer period of time compared to the 1-day in-person workshop, which the majority of participants felt was adequate. However the longer duration required for participation may be perceived as a disadvantage by some. The standard JLA methodology for stakeholder involvement in health care research prioritization, including a final in-person workshop, has been implemented to date in more than 20 chronic diseases, including prior work by our group to identify research priorities for patients on or nearing dialysis [22]. A recent systematic review on research priority setting in kidney disease identified 16 studies, only 4 of which involved patients in the prioritization process [23]. The studies cited in this review used a variety of methods to elicit research priorities, none of which were described in detail nor used an electronic or online collaborative platform to engage stakeholders, and none of which were evaluated in a randomized trial such as this one.

Wiki-like tools have been applied in other collaborative health care-related activities involving patients and other stakeholders. Gupta et al. included patients and clinicians in a wiki-inspired collaborative design of an asthma action plan (WikiBuild), where groups of 14 participants contributed to the action plan’s content and design over a 1-week period [16]. Other studies examining the application of wiki-based tools in patient-centered research, including the development of clinical practice guidelines [24] and patient information leaflets [25], have found these tools to be promising alternatives to more traditional approaches. In contrast to our study, these other studies generally involved input from a large numbers of patients (>300) and lack the personalized and multi-stakeholder interactions endorsed by the JLA format.

Potential advantages of an online wiki-inspired approach for collaborative research prioritization include flexibility in participation, lack of travel requirements limiting participant eligibility, and lower costs [16]. A particular advantage is the ability to include those whose illnesses may preclude easy travel; for example, one patient in the wiki-inspired group in our study was admitted to hospital during the study period and participated in the process while in hospital. The complex group dynamics often seen with in-person techniques can lead to the development of status hierarchies that favour clinicians over patients and caregivers [19, 26, 27], which could limit the openness of individual contributions to the discussion and influence the final product [28], although our qualitative results would suggest that was not the case in our study. Although neither the wiki group nor the in-person workshop group indicated the presence of overly influential individuals, wiki-inspired participants indicated the ability to contribute honestly and explicitly in this format.

Although 61 % of wiki-inspired participants responding to the post-intervention questionnaire felt that the chat feature was useful for explaining their views and preferences, many expressed concerns that this format did not allow for as comprehensive a discussion and understanding as is possible with face-to-face interactions. Gupta et al. described similar participant concerns for the wiki online chat, as well as the lack of individual accountability for changes made [16]. Other barriers to the adoption of wiki-inspired tools for patient engagement activities described include concerns regarding suitability of the format to its objectives, poor participation rates, and technical challenges, among others [16, 24, 25]. It is unclear whether patients with poor health literacy or cognitive impairment would be able to participate in a wiki-inspired priority setting process, and this is an area for future work. The participant feedback provided during our trial highlights the need to refine the online collaborative tool, and the chat feature in particular, to ensure participants feel engaged and able to contribute meaningfully to the process.

Our study is one of the first to describe the application of a wiki-inspired tool to a research prioritization exercise, with a randomized comparison of methods for eliciting input from a diverse group of stakeholders to determine research priorities. The results, however, should be interpreted in light of the study limitations. The requirement for participants to have regular access to a computer and the Internet may have limited eligible participants and thus the generalizability of findings. However, Internet access is increasingly common among patients with chronic disease (up to 70 % of patients with chronic illness are Internet users) [29], and approximately half of patients with advanced CKD access the Internet to obtain health information [30, 31]. Further, little evidence exists to guide the evaluation of stakeholder engagement with such consensus-driven processes [32]. While quantitative and written responses on the post-intervention survey provide valuable information, additional insights could be gained through participant interviews evaluating individual experiences with the interventions. Finally, 6 of the patient and caregiver steering group members participated in the NGT in-person workshop, and were not part of the randomization process. Although these steering group members were involved in the earlier stages of the CKD priority setting partnership (ie identification of research priorities through national survey and refinement of these priorities into a top 30 shortlist), the final ranking of the top 10 priorities was a collaborative process involving all stakeholders participating in the workshop. All participants were encouraged to contribute to the final ranking process, and the top 10 priorities reflect group consensus rather than the views of any one individual.

## Conclusions

Involving patients and other stakeholders in determining research priorities has the potential to enhance the quality and relevance of research for end users. In our randomized controlled trial of patients, caregivers, clinicians and policymakers, we found that an online collaborative wiki-inspired process identified different top ten CKD-related research priorities compared with an in-person workshop, and participants in the wiki group were less likely to feel engaged and satisfied. We are unable to determine if the differences in research priorities identified were due to the different processes or differences in the groups themselves, as priorities may vary by group irrespective of the process used to establish them. This is important to address in future work. Modifications to the wiki tool, including optimization of a communication feature to improve the ability of participants to communicate their views and preferences, need to be designed and evaluated before a wiki process can be considered a feasible alternative to an in-person workshop for engaging patients in determining research priorities.

## Additional files

Additional file 1: Table summarizing methods used for assessing primary and secondary outcomes. (DOCX 104 kb) Additional file 2: Figure demonstrating usability responses from the wiki-based group on the post-intervention questionnaire. (DOCX 120 kb)

## Abbreviations

CI: Confidence interval
CKD: Chronic kidney disease
CONSORT: Consolidated Standards of Reporting Trials
eGFR: Estimated glomerular filtration rate
JLA: James Lind Alliance
NGT: Nominal group technique
PCORI: Patient-Centered Outcomes Research Institute
SPOR: Strategy for patient oriented research
