# Supplementary material for: A randomized controlled trial comparing in-person and wiki-inspired nominal group techniques for engaging stakeholders in chronic kidney disease research prioritization
Source: BMC Med Inform Decis Mak. 2016 Aug 24;16(1):113. doi: 10.1186/s12911-016-0351-y (PMC4995639; doi:10.1186/s12911-016-0351-y)
Supplement: Additional file 1: — Table summarizing methods used for assessing primary and secondary outcomes. (DOCX 104 kb) [file 12911_2016_351_MOESM1_ESM.docx]

**Additional File 1. Summary of outcomes assessed and methods of measurements**

| **Outcome** | | | **Method of Measurement** |
| --- | --- | --- | --- |
| *Primary Outcome* | | |  |
|  | Pairwise agreement between the two groups’ top 10 ranked priorities* | | Spearman’s correlation and descriptive comparison of the final top 10 priorities lists recorded upon conclusion of each process |
| *Secondary Outcomes* | | |  |
|  | Participant satisfaction and engagement with each process | | Electronic questionnaire assessing participant satisfaction and engagement with the process and format. Responses were graded on 5-point Likert scale (1=strongly disagree, 5=strongly agree). Survey was available for 2 weeks following each process. Opportunity for written feedback was available in each section of the survey. Participant observation data was also collected during the in-person workshop using a template to report verbal and physical behaviours. |
|  | Wiki usability (content, features, accessibility) | | Electronic questionnaire assessing wiki usability aspects administered to wiki group only. Responses were graded on 5-point Likert scale. Opportunity for written feedback was available. |
|  |  | Page requests | Date, time and number of requests made by each participant to load the login and/or ranking pages. |
|  |  | Direct changes to list | Number of times each participant moved a priority to a new position (for example, moving ranked item #10 into position #5 was considered one direct change). |
|  |  | Chat comments | Date, time and number of contributions each participant made to the chat feature. The full chat transcript was also available. |
|  |  | Technical issues | All attempts by participants to contact the research team for technical assistance were recorded, including the problem and resolution status. |
|  | Time to undertake each process | | Time requirements for each process compared descriptively between groups. For the in-person group, this included the workshop and any travel time to attend. For the wiki group, this included the 3-week period of tool availability, as participants could access the site at any time of day at their convenience. |
|  | Costs to undertake each process | | Overall costs of completing each process compared descriptively between groups. Costs of the in-person group included transportation, accommodations, meals, facilities rental, materials, and training and use of facilitators and research assistants. Costs of the wiki group included tool development and programming, and technical support available throughout the process. |

*Both groups ranked the top 10 research priorities; the in-person group was also asked to rank priorities 11-30
