# Supplementary figures and images for: A randomized controlled trial comparing in-person and wiki-inspired nominal group techniques for engaging stakeholders in chronic kidney disease research prioritization
Source: BMC Med Inform Decis Mak. 2016 Aug 24;16(1):113. doi: 10.1186/s12911-016-0351-y (PMC4995639; doi:10.1186/s12911-016-0351-y)

**Additional File 2. Wiki usability responses on post-intervention questionnaire**

**
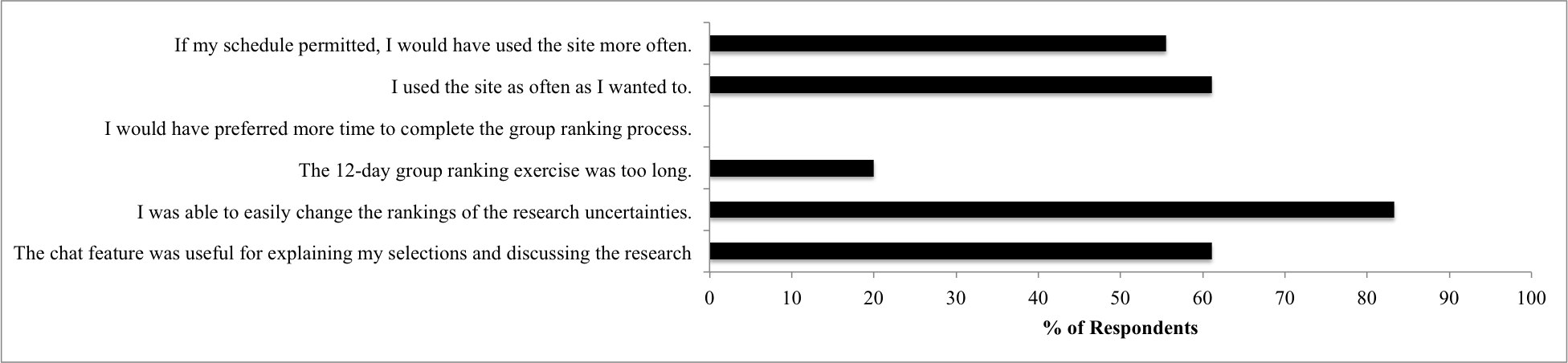
**

Supplement: Additional file 2: — Figure demonstrating usability responses from the wiki-based group on the post-intervention questionnaire. (DOCX 120 kb) [file 12911_2016_351_MOESM2_ESM.docx]
